# Comparison of the Electrical Response of Cu and Ag Ion-Conducting SDC Memristors Over the Temperature Range 6 K to 300 K

**DOI:** 10.3390/mi10100663

**Published:** 2019-09-30

**Authors:** Kolton Drake, Tonglin Lu, Md. Kamrul H. Majumdar, Kristy A. Campbell

**Affiliations:** Department of Electrical and Computer Engineering, Boise State University, Boise, ID 83725-2075, USA

**Keywords:** chalcogenide, electrochemical metallization cell, electrochemical metallization (ECM), ion conduction, memristor, self-directed channel (SDC)

## Abstract

Electrical performance of self-directed channel (SDC) ion-conducting memristors which use Ag and Cu as the mobile ion source are compared over the temperature range of 6 K to 300 K. The Cu-based SDC memristors operate at temperatures as low as 6 K, whereas Ag-based SDC memristors are damaged if operated below 125 K. It is also observed that Cu reversibly diffuses into the active Ge_2_Se_3_ layer during normal device shelf-life, thus changing the state of a Cu-based memristor over time. This was not observed for the Ag-based SDC devices. The response of each device type to sinusoidal excitation is provided and shows that the Cu-based devices exhibit hysteresis lobe collapse at lower frequencies than the Ag-based devices. In addition, the pulsed response of the device types is presented.

## 1. Introduction

Self-directed channel (SDC) memristors are a type of chalcogenide-based electrochemical metallization (ECM) device [1,2,3,4,5,6,7] in which it is posited that re-usable and irreversible ion-transport channels are formed within the active chalcogenide layer during the first write operation [6,7]. The persistence of these channels, even after the device is cycled between high and low resistance states, is considered the largest factor responsible for consistent SDC device state switching [6]. ECM devices of many different material types, ranging from oxides, to chalcogenides, typically using Ag or Cu as the ion source, are lauded as having the highest likelihood for success in next generation non-volatile memory, neuromorphic computing, and space applications where a robust, radiation hardened, and temperature tolerant device is desirable [1,2,3,4,6,7,8,9,10,11,12,13,14,15,16,17,18,19,20]. Investigation of ECM device operational theory is ongoing since device improvement and good application-based device design requires a closer understanding of how the devices work. 

Recently, there has been a trend in the literature to classify all ECM device types as conductively bridged random access memory (CBRAM) devices [4,21]. This generalization is in conflict with the earlier literature where CBRAM was used to describe a specific device type in which a conductive filament is formed through a solid solution, e.g., Ge_x_Se_1-x_ (or Ge_x_S_1-x_) where x < 0.33 [10,11,12,13,22,23,24,25,26,27,28,29,30,31]. These devices have also been referred to as programmable metallization cell (PCM) devices [13,26,27,28,29]. Now, the CBRAM designation is used synonymously with ECM [4] and over the years has included oxide-based materials as well as organic materials [32] and even BN films [33]. We classify the SDC memristor as an ECM device but remove it from the general classification of CBRAM for three reasons. First, the description of “GeSe-based CBRAM” is currently associated with a doped solid solution GeSe-Ag system [25,28,30]. The SDC does not contain a doped solid solution; the device does not require Ag doping, nor allow doping [7]. Unlike the GeSe-Ag CBRAM device, the SDC device structure contains material layers meant to store metal ions (the SnSe layer or other metal-chalcogenide layer—see [7] for experimental data and a discussion of the effects of the metal on SDC operation) and to facilitate fast switching; these are not present in the GeSe-Ag solid solution-based device.

Second, the fabrication methods, operation, temperature tolerance, device switching consistency, and longevity of the SDC are significantly different from the GeSe-Ag CBRAM, so separation prevents confusion between the two types of ECM devices. The SDC device can withstand higher fabrication temperatures than other typical chalcogenide-based ECM devices, which gives it more flexibility for manufacturing in a commercial facility. SDC devices have also been shown to operate at high temperatures (150 °C) for an extended time without performance degradation, and have been shown to function normally after reaching high temperatures (at least 250 °C) [6]. The SDC device fabrication is simple, requiring no photodoping or thermal annealing for incorporation of oxidizable metals. The device materials can all be sputter deposited in-situ making thin film deposition simpler as well as protecting the device material layers from oxygen and detrimental water exposure [34]. During the fabrication steps, the chalcogenide film stack is never exposed to photolithography chemicals or solvents; the final device etch step is performed by ion milling (no chemical etching), thus further preventing any water or oxygen exposure to the device active layer. The Ag-based SDC device longevity has been physically measured over a time of more than 10 years (see Supplementary Material, Figure S1). 

Third, and most importantly, it is a working hypothesis that the SDC operation requires a separate metal chalcogenide layer and an amorphous active layer, such as Ge_2_Se_3_, which contains thermodynamically unstable homopolar bonds (such as the Ge-Ge bonds that are present in the SDC device Ge_2_Se_3_ active layer). Channel formation then occurs through an irreversible chemical reaction between the device material layers upon the first programming event [7,35]; the combination of these layers will reactively generate permanent channels, i.e., Ag or Cu ion transport routes, through the SDC device Ge_2_Se_3_ active layer via a chemical reaction preferentially with the Ge-Ge bond sites. Once the channel is formed, it is permanent under similar operating conditions, with the device state change depending on Ag or Cu ion movement within the established channel. There is no “dissolution” of randomly ordered conductive filaments into the material matrix film which is the hallmark of CBRAM [36,37]; there is simply movement of metal ions within a well-defined transport route. Ag or Cu ions can move into or out of the channels, corresponding to a write or erase for modification to a lower or higher resistance. The channels enable more consistent and predictable switching within a device as well as between different devices compared with the other chalcogenide-based ECM device types. After channel formation, the SnSe layer can be considered an intermediate layer, or ‘stepping stone’ for oxidation of Ag or Cu, and storage of metal ions. The formed channels assist the device in fast and consistent switching since it allows formation and storage of oxidizable metal ions instead of overcrowding and saturating the active glass layer. The desired morphology of the SnSe layer is thus one that is disordered, with a large surface area for Ag or Cu (and their ions) to react with SnSe [7].

A similar approach has recently been used in amorphous carbon (a-C) ECM devices which use Ag as the oxidizable metal [38]. In this case, a layer of AgInSbTe was used to buffer the a-C film from oversaturation of Ag, as well as to provide a location for Ag-ion storage. Similarly to the SDC device compared with and without the SnSe layer [7], these a-C devices exhibited highly uniform switching, high cycling endurance, and fast switching times only in the presence of the storage layer. 

Even though there are ECM devices with materials systems that appear similar to the SDC due to the chemical elements present in the device [22], the extreme differences in device operation, fabrication and stability justify placing the SDC memristors in their own ECM subcategory as a “self-directed channel”.

In this work, we compare the direct current (DC) (quasi-static) and pulse electrical response of Ag and Cu SDC memristors as a function of temperature from 6 K to 300 K, and discuss the device stability under various programming conditions. 

## 2. Materials and Methods 

### 2.1. Device Structure and Fabrication

**Figure 1:**
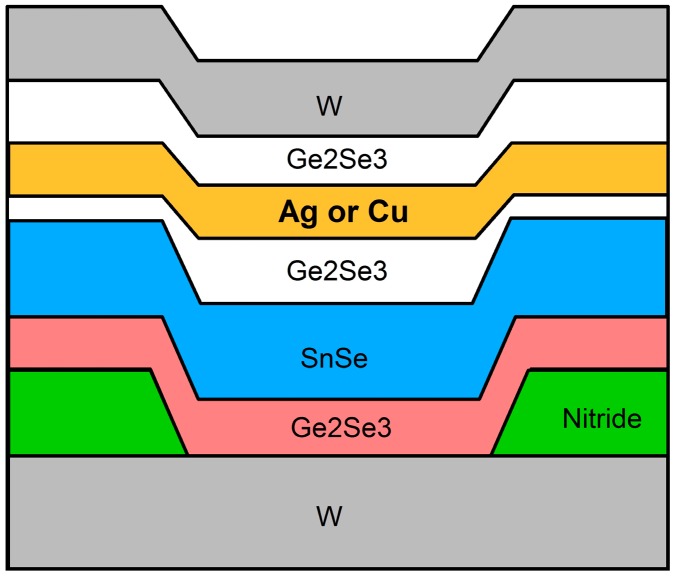
Self-directed channel (SDC) device structure described in [6]. The target layer thicknesses were (from bottom to top): Ge_2_Se_3_ (300 Å)/SnSe (800 Å)/Ge_2_Se_3_ (150 Å)/Ag (500 Å)/Ge_2_Se_3_ (100 Å)/W (400 Å). The top three layers below the W top electrode, corresponding to Ge_2_Se_3_/Ag/Ge_2_Se_3_, mix during fabrication, becoming one conductive layer.

Devices were fabricated in the Idaho Microfabrication Laboratory at Boise State University on 100 mm p-type wafers in a stacked layer structure (Figure 1). The device size is defined by the bottom electrode contact area and is 2 μm in diameter. The devices were fabricated with either a Ag or Cu layer as the mobile ion source layer. The active layer, responsible for device resistance switching is the bottom Ge_2_Se_3_ layer in contact with the bottom W electrode, within the nitride opening. The details of the purpose of each thin film layer have been described previously [6] and a full discussion can be found there. In brief, the SnSe layer assists in the formation of the self-directed channel within the active layer and acts as a cation storage layer. The two Ge_2_Se_3_ layers surrounding the Ag or Cu layer enable thin film adhesion and photolithography. The active switching layer is the bottom Ge_2_Se_3_ layer.

Prior to thin film deposition using an AJA International ATC Orion 5 UHV Magnetron sputtering system, the wafers were sputtered with Ar^+^ to prepare the bottom electrode surface. This was followed by *in-situ* sputter deposition of all of the remaining device layers, including a top W electrode capping layer. *In-situ* deposition of layers was performed to minimize the potential for detrimental water vapor on the device [34]. Final device etching was performed with a Veeco ME1001 ion-mill (Veeco, Plainview, NY, USA).

### 2.2. Electrical Measurements

Temperature control and sample probing was performed using a Lake Shore CRX-4K probe station, two Lake Shore Model 340 temperature controllers (Lake Shore Cryotronics, Inc., Westerville, OH, USA), a SHI RDK-408D2 closed-cycle refrigerator (Sumitomo (SHI) Cryogenics of America, Inc., Allentown, PA, USA) with a controlled temperature range of 5 K to 400 K, and an RC-EM10-208230-60 CE liquid helium recirculating chiller. Lake Shore ZN50R alumina ceramic probe cards with 25 µm Tungsten tips were used for measurements. Probe cards were anchored to the sample stage with copper braiding to ensure temperature equilibration between stage and probe. Vacuum was maintained and monitored with a Varian V-81 turbo pump.

DC (quasi-static), sinusoidal excitation, and pulsed measurements were made using a Keysight B1500A Semiconductor Parameter Analyzer equipped with two Waveform Generator/Fast Measurement Units (WGFMUs) (Keysight, Inc., Santa Rosa, CA, USA). The WGFMUs allowed direct measurement of the current through the device during testing without external circuits or current limiting series resistors. The sweep rate for a DC measurement depends on the voltage and current ranges used, which are varied depending upon the sample measurement temperature and write/erase measurements; however, for all measurements the sweep rate is in the range of 0.14 to 0.2 V/s (switching voltage vs sweep rate is shown in the Supplementary Material, Figure S2).

At least 10 unique devices were measured at every temperature, for both the Ag and Cu-based devices. Three trials of temperature measurements were performed over a two year period after wafer fabrication. During the storage periods, the samples were maintained in the dark, at ambient temperature. Since the effect of cold temperature on the devices was unknown at the start of the experiment, wafer pieces were measured in the order of decreasing and increasing temperature. It was determined that the temperature order of measurement did not influence the measurement outcome. Therefore, for the experimental data provided in this work, the samples were brought to a base temperature of 6 K and equilibrated for 30 min prior to commencing measurements. The temperature was raised for each subsequent temperature measurement, with an equilibration of at least 30 min at each temperature prior to the measurement.

All DC sweep measurements consisted of the sequence: Write 1-Erase-Write 2-Read. The Write sweeps applied a positive potential to the device top electrode and used a 10 × 10^−6^ A compliance current. The Erase sweep applied a negative potential to the top electrode; a 10 × 10^−3^ A compliance current was applied. A +20 mV Read sweep was applied to the top electrode to read the final written resistance state after the Write 2 step (Read). In all measurements, the bottom electrode was maintained at ground and the top electrode potential was varied. For the Ag devices, the Write 1 and Write 2 sweeps were performed over the range of 0 to 3 V for T ≥ 150 K, and 0 to 5 V for T < 150 K. For the Cu devices, the Write 1 range was 0 to 3 V for T ≥ 140 K and 0 to 5 V for T < 140 K. The Erase voltage ranges for each type of device were the same, except in the negative potential direction.

Prior to the first (Write 1) sweep, all devices tested were in a pristine (never previously tested) state. Table 1 summarizes the sweep measurement and Read voltage for each resistance type. The conductance of a device was calculated from 1/R, where R is the measured resistance.

## 3. Results

### 3.1. DC (Quasi-Static) Measurements

**Figure 2:**
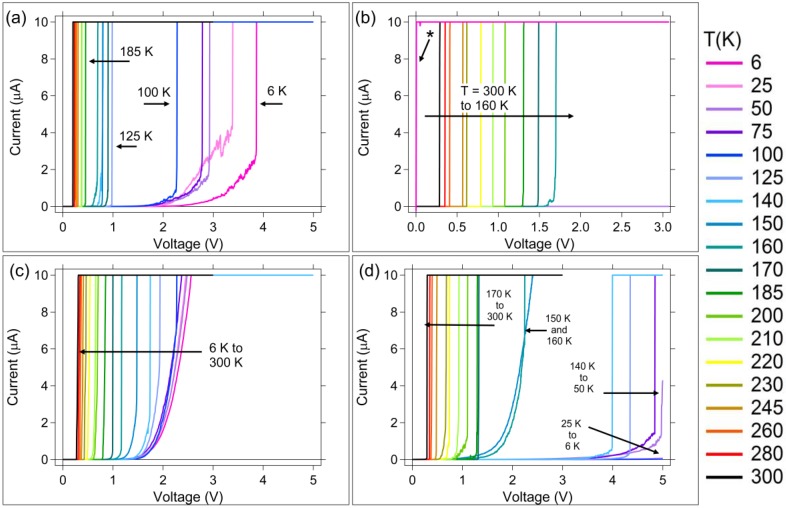
Representative Write I–V curves as a function of temperature. Ag devices: (**a**) Write 1; (**b**) Write 2. Cu devices: (**c**) Write 1; (**d**) Write 2. The * in (b) corresponds to I–V curves of broken (shorted) devices. Note: the Write 1 sweep voltage maximum for measurements below 150 K was 5 V, compared to 3 V used for T ≥ 150 K.

**Figure 3:**
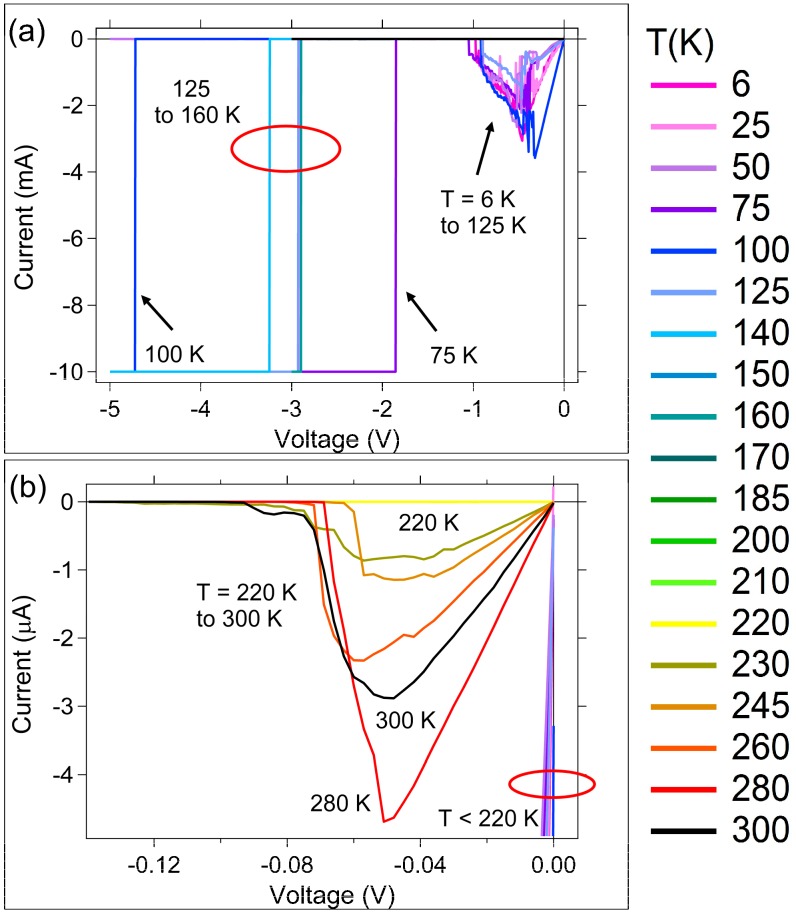
Ag device representative Erase I–V curves for all temperatures (see legend). (**a**) Full scale view; (**b**) expanded low I–V region.

**Figure 4:**
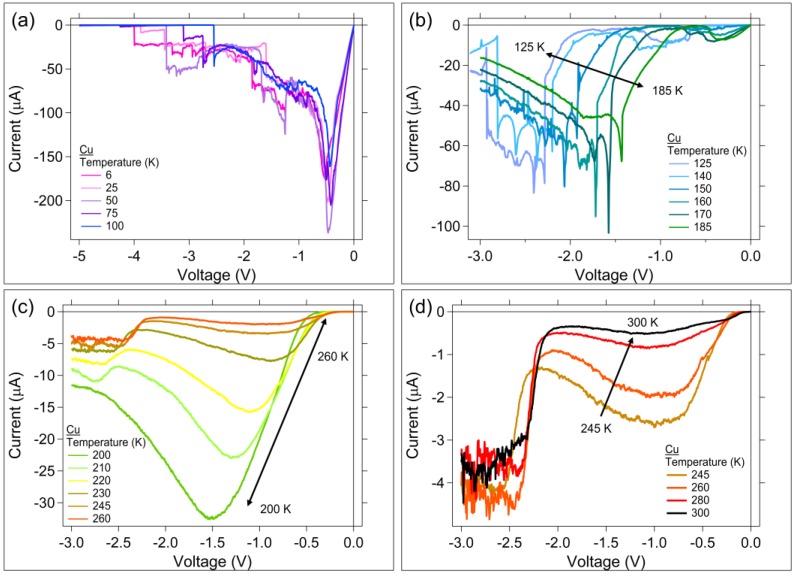
Cu device erase regions for (**a**) 6 K ≤ T ≤ 100 K; (**b**) 125 K ≤ T ≤ 185 K; (**c**) 260 K ≤ T ≤ 200 K; and (**d**) 245 K ≤ T ≤ 300 K.

Representative DC I–V measurement curves for each measurement sequence at each temperature are shown in Figure 2, Figure 3 and Figure 4. Write 1 and Write 2 curves are shown in Figure 2a,b for Ag, and Figure 2c,d for Cu. The Erase sweeps are shown in Figure 3a,b for Ag devices and Figure 4a–d for Cu devices.

The Write 1 sweep is the first time voltage is applied to a pristine device. This measurement can therefore provide the initial device resistance when measured at +20 mV during the Write 1 sweep. The Write I–V curves in Figure 2 are typical for an SDC device. The Write sweep starts at 0 V and the potential is increased until the device transitions to a low-resistance state, at which point the current reaches the compliance current. In the pristine state, Ag-based SDC devices initially have a very high resistance (GΩ range), and exhibit either an instantaneous increase in current to the compliance value during the Write 1 sweep, or an exponential rise in current with applied voltage, depending upon temperature. The exponential increase in current is present in the low temperature Write 1 sweeps for Ag and Cu (Figure 2a,c), and Cu Write 2 sweeps (Figure 2), but is absent in the Write 2 sweep for Ag (Figure 2b).

The Write 1 I–V sweeps, Figure 2a,c for both device types, show that devices switch with an increasing switching voltage as the temperature is reduced. At 6 K, the Ag devices switch at approximately 4 V. However, as can be seen in the erase sweeps (Figure 3a,b), not all of the Ag devices that switched at low temperature can erase. These devices appear as ‘shorts’ on the Write 2 I–V sweeps (denoted by * in Figure 2b). The devices that were switched at low temperature could not be cycled back, despite the large compliance current on the Erase sweep of 10 × 10^−3^ A, compared to 10 × 10^−6^ A on the Write sweep. Measurements upon return to room temperature indicated the devices were destroyed. 

An Erase sweep corresponds to application of a reverse polarity potential to the top electrode. Since the erase occurs after the SDC device was written, the low potential region of the Erase I–V curve for the Ag device (Figure 3a,b) shows a mostly linear behavior up to a peak current value, as expected for a device programmed into a resistive state. Beyond the peak current voltage, the device experiences negative resistance as it transitions towards a high resistance state. Erase sweeps are shown in an expanded view in Figure 3b for Ag devices, in which only the data for T ≥ 220 K is in view since the peak current is 1000 times lower than for T < 220 K (low temperature, higher currents, are dominant in the full-scale view in Figure 3a). The current required to erase the Ag devices that switched at temperatures below 150 K is in the mA range, which is three orders of magnitude higher than under room temperature conditions (Figure 3b). For the temperatures between 140 K ≤ T ≤ 220 K, the devices that were switched during the Write 1 sweep (Figure 2b) did not latch the low resistance state, so no Erase current is measured (it is within the noise of the instrument and too low to observe in Figure 3a or Figure 3b).

It is reasonable to expect that not all Cu or Ag ions generated during the Write sweep get reduced within the duration of the Write sweep. It is anticipated that when the voltage is removed, there is a concentration of ions still within the active layer channels that are oxidized. In other words, not all ions generated during the Write sweep will reach a conductive contact point, directly or indirectly, with the bottom electrode during the measurement. These ions can remain within the channel, diffusing towards a more energetically stable location in the channel or the SnSe layer. As temperature is reduced, the possibility of the ions diffusing is reduced, making it more likely that the low temperatures yield more excess ions within the devices.

The low temperature Ag device Erase I–V curves (Figure 3a) are much different than the curves for higher temperatures (Figure 3b). For the 300 K to 230 K Erase sweep data (Figure 3b) there is a maximum current peak at approximately −0.05 V, and a linear slope leading up to this peak from 0 V. These data are consistent with the rupture, or loss of contact, of a conduction path between the electrodes. In contrast, a broad peak in the Ag device Erase sweeps (Figure 3a) at temperatures below 150 K occurs between −0.3 to −0.5 V. This voltage range is 10 times greater than the potential needed to break conductive contact between electrodes for 230 K ≤ T ≤ 300 K. Cyclic voltammograms for Sn-Ag systems [39] can show broad peaks between −0.2 V and −0.8 V, depending upon the concentrations of Ag and Sn in the system, and formation of a Sn-Ag alloy. The observed broad peak for T < 150 K is within the range observed in cyclic voltammetry of systems comprising the formation of Sn-Ag alloys [39]. Since Sn can participate in redox reactions during the channel formation and subsequent switching cycles, it is possible that the maximum sweep voltage (5 V) during the low temperature Write sweep is high enough to oxidize Sn. Any excess Ag^+^ that remained upon removal of the Write sweep potential would still be present within the device, with ion diffusion occurring much slower at the low temperatures. Reduction of the excess Ag^+^ is therefore possible during the Erase sweep, and would appear more prominently at low temperatures where higher ion concentration is expected. Interestingly, the low temperature Write 1 I–V curves exhibit an exponential increase in current, not observed in the higher temperature curves (Figure 2a). This, in combination with the Erase peak occurring within a Sn-Ag alloy redox potential range, could indicate Sn involvement in switching. With the Ag consumed in an alloying reaction with Sn, Ag would no longer be available for ion movement during programming. A Sn-Ag alloy could produce a permanently conductive pathway within the channel. The Ag devices in the low temperature range were readily damaged by application of potentials higher in magnitude than −1 V during the erase, as seen by the sharp transitions to compliance current on the Erase sweep (Figure 3a). In order to erase the devices that did write at low temperature, currents as high as almost 4 mA were needed. The formation of a Sn-Ag alloy may be why the devices that switch at low temperature are ‘shorted’ and no longer function.

Similarly to the Ag case, at low temperatures, the Cu devices required higher applied potentials to switch; the Cu device threshold voltage increased as the temperature decreased (Figure 2c). If the only redox consideration were Cu, it could be concluded that the oxidation of Cu during the Write will occur first through Cu → Cu^2+^, then as voltage is further increased, it would go directly from Cu → Cu^+^. Therefore, it would be expected that for the cold temperature Write sweeps, the generation of Cu^+^ would be possible due to the increased Write Sweep potential. It is further expected that there would be excess Cu^+^ and Cu^2+^ in the device upon removal of the Write potential; at cold temperatures, the diffusion of the ions would be significantly reduced, thus keeping a higher concentrations of ions within the channel. Upon application of an Erase sweep, these excess ions could be reduced. Reduction of the Cu ions would appear in the Erase sweep as a reduction of Cu^+^ → Cu at a potential near −0.5 V. A peak around −0.45 V is observed for the Erase sweeps between 5 to 100 K, Figure 4a. This is not observed in the other Erase temperature ranges, where the Write 1 sweep voltage maximum was 3 V, instead of 5 V, and therefore was likely not high enough to achieve the Cu → Cu^+^ oxidation. However, as in the case for Ag, a contribution due to Sn redox reactions cannot be ruled out.

The Erase sweeps for the Cu sample have at least four temperature regions with differing I–V curve characteristics (Figure 4a–d). Since Cu can oxidize during the Write sweep to both Cu^+^ and Cu^2+^ depending upon the magnitude of the applied potential, it is expected that the Cu device Erase I–V curves could be more complicated than the Ag device Erase curves, and potentially have between two and three peaks corresponding to different Cu ion reduction potentials. Based on cyclic voltammograms [34] it is expected that the peak near the lowest magnitude Erase potential corresponds to a Cu^+^ → Cu reduction. The next highest potential would correspond to a Cu^2+^ → Cu reduction, and the highest potential to the Cu^2+^ → Cu^+^ reduction. However, Cu can also form an alloy with Sn [40]. Therefore, the observed peaks may be complicated by multiple redox reactions of Cu and Sn. 

**Figure 5:**
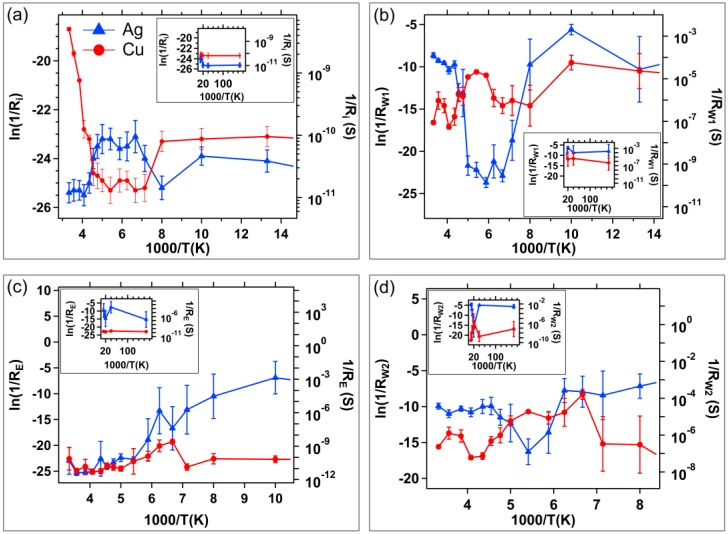
Average conductance versus inverse temperature for (**a**) initial resistance, R_i_; (**b**) written resistance after the first write sweep, R_W1_; (**c**) erased resistance, R_E_; and (**d**) written resistance after the second write sweep, R_W2_. In each graph, the Cu device data is represented by circles; Ag devices as triangles. The inset of each graph is the extension of the data into the coldest temperature region. Error bars are one standard deviation.

The Erase peak potentials for the Cu-based devices have a significant temperature dependence above 125 K. Figure 4b–d show the Erase sweeps for temperatures from 125 K to 300 K. For 185 K ≥ T ≥ 125 K (Figure 4b), there are multiple small peaks on I-V curves between −3 V to −1.4 V. There are also low amplitude broad peaks between −0.3 V to −1.25 V. In all cases, the I-V curves exhibit a temperature dependence, with peak shifting to lower voltage as the temperature is increased. The higher voltage region peaks exhibit an increasing number of sharp peaks as the temperature is reduced. Similar sharp peaks have been observed in a Cu-Sn alloy reaction [40]. Within the temperature range 260 K ≥ T ≥ 200 K (Figure 4c), the largest Erase peak voltage (at approximately −1.5 V for the 200 K trace) and current at the peak, has a significant dependence on temperature, with the peak voltage and current decreasing with an increase in temperature. This is also the temperature range where the Cu device exhibits a negative slope in the ln(1/R_W1_) vs 1000/T plot (Figure 5b). The peaks that occur between −2 and −3 V in Figure 4 could correspond to a Cu^2+^ → Cu^+^ reduction due to excess Cu^2+^ present in the channel following the Write sweep [34].

The average initial, written, and erased resistances as a function of temperature are provided in Figure 5, plotted as ln(1/R) vs 1000/T. Error bars correspond to 1 standard deviation. The lowest temperature region is displayed in the inset of each plot.

As discussed, and apparent in the Write 2 sweep, Figure 2, and the Erase sweep in Figure 3, Ag devices did not always switch at low temperatures. The inset of Figure 5c shows this clearly: instead of erasing to high resistance, R_E_ is a similar magnitude to R_W1_ and R_W2_ at temperatures below 125 K. This indicates that the Ag devices were damaged or permanently altered during the Write 1 sweep. The Cu devices clearly erased to high resistances over the entire temperature range.

**Figure 6:**
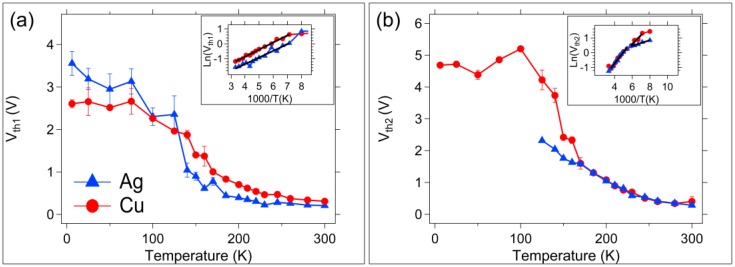
Average write threshold voltages as a function of temperature. (**a**) First Write, V_th1_; (**b**) Second Write, V_th2_. The inset in (a) and (b) is the Ln(V_th_) vs 1000/T plot showing the Arrhenius behavior for the threshold voltage of both device types. Error bars represent one standard deviation.

The switching (threshold) voltage, V_th_, for a Write voltage sweep is identified as the potential at which a large current jump is initiated towards the compliance current value. These switching voltages were determined for each I-V trace of the Write 1 and Write 2 sweeps and have been plotted as a function of temperature in Figure 6 as V_th1_ and V_th2_, respectively. There is an exponential relationship between V_th_ and T for both threshold voltages above 150 K. This is clear in the inset graph which plots Ln(V_th_) vs 1000/T and the corresponding linear fit. No data is available for V_th2_ for the Ag devices operated below 150 K due to low temperature operational damage (Figure 6b).

### 3.2. Sinusoidal Excitation and Pulsed Response

**Figure 7:**
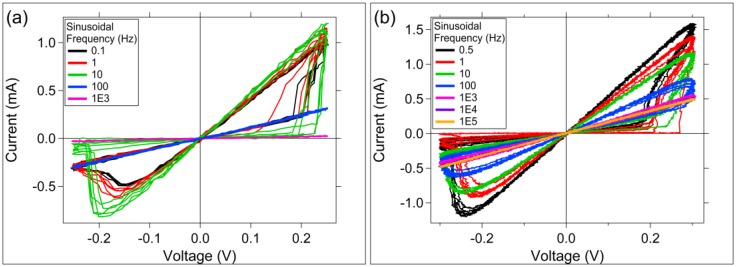
Cu and Ag device response to a sinusoidal input with varying frequency. (**a**) Cu device; (**b**) Ag device. T = 300 K. Six cycles at each frequency are shown.

A sinusoidal input signal was applied to each device type, and the device response as a function of frequency of the input signal was measured (Figure 7). Both device types exhibit the characteristic fingerprint of memristors, a pinched hysteresis loop, under sinusoidal excitation (Figure 7) [41]. In both cases, the device response is pinched at the origin, and the hysteresis lobe area is decreased to zero as the input signal frequency is increased. Cu devices (Figure 7a) display flattened hysteresis lobes at a low frequency of 100 Hz, whereas for the Ag devices (Figure 7b) this occurs at 10 kHz.

**Figure 8:**
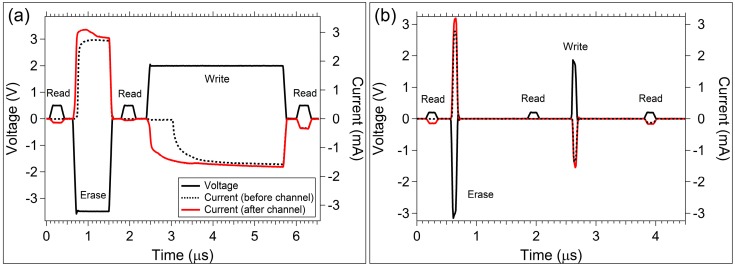
Cu and Ag device response to a programming pulse sequence. The solid black trace corresponds to the applied voltage pulse sequence. The dashed lines correspond to the current measured during the voltage pulse sequence for a pristine (before channel formation) device. The solid red line corresponds to the current measured during the next applied pulse sequence after channel formation. (**a**) Cu device; (**b**) Ag device. T = 300 K.

The pulse response is measured by applying a programming voltage pulse sequence (as labeled in Figure 8) to the memristor. The response of the memristor is determined by the current measured through it during application of the voltage pulse. The current measurement is opposite polarity from the voltage pulse sequence due to the instrument set up; a negative current is measured when voltage is positive. An adjustment of the data to the correct sign of current is not made, since this allows current and voltage data to be displayed on the same graph (right and left axes, respectively) with minimal interference.

The response of Cu devices to the programming pulse sequence is provided in Figure 8a. A pristine Cu device (never switched previously, but from the R_i_ data in Figure 5a, does appear to have some Cu diffused into the active layer) was tested. The current through the pristine device during the voltage pulse sequence is given by the dashed line trace in Figure 8a. A Read pulse was applied first; the current response during the Read pulse is too low to observe on the mA scale, indicating that R_I_ was higher than 10 MΩ. The second Read pulse also shows no measureable current, indicating the device is still in a high resistance state following the Erase pulse (as anticipated). Given that the Cu device was pristine, the self-directed channel has not yet been formed in the active layer at this point in the pulse sequence (i.e., prior to the Write pulse). Channel formation happens during the first Write pulse. Note that there is approximately 500 ns delay from the initiation of the Write pulse and the current response. This delay is likely due to oxidation of Cu, and the chemical reaction taking place within the SnSe layer and active layer to form the channel. The measured current through the device during the final Read pulse indicates that the device was written to a low resistance state during the Write pulse.

A second pulse sequence was applied to the same Cu device two minutes after the previous measurement. The current response to this second sequence is given by the red trace in Figure 8a. The device was still in a low resistance state from the previous measurement (as indicated by the current through the device during the first Read pulse of the second applied pulse sequence, red trace Figure 8a). However, the amplitude of the current response during the Read pulse is lower than the Read current measurement at the end of the first pulse sequence, indicating that the Cu device exhibits a drift in the programmed resistance state. Following the Erase pulse of the second pulse sequence, the Read pulse indicates the device resistance was increased successfully. Application of the Write pulse on this second pulse sequence does not have the delay in device response that was observed in the first Write pulse, as expected since the channel was formed on the prior pulse sequence, and storage of Cu is presumed to be present in the SnSe layer.

The Ag devices, Figure 8b, did not exhibit the large delay in initial switching during channel formation. In this case, if there is a delay during channel formation, it is beyond the resolution of the pulse timing.

## 4. Discussion

Even though Figure 5 provides the measured conductance as a function of temperature, it must be noted that while the conductance plots in Figure 5 are plotted in an Arrhenius form (ln(1/R) vs 1/T), these data are not typical conductance vs temperature measurements where one could determine conduction activation energies accurately, or reliably investigate conduction mechanisms. The conductance value at each temperature is determined from the resistance that the device achieved upon switching *at* a particular temperature. There are many factors that go into device switching at each temperature for the SDC device. Some examples include the temperature dependence of the chemical reaction between Ag or Cu and the SnSe layer and induced reactions in the active layer; movement of mobile ions through a variable-stiffness glass network; constricted channel for ion motion (e.g., due to cold temperature volume contractions); and the typical DC conductivity mechanistic concerns (e.g., Fermi energy level and dominant electron conduction mechanism at each temperature [42,43,44]). It could also be reasoned that even programming a set of devices to a state value, and then subjecting the devices to a set of varying temperatures and measuring conductivity could also confound the mechanism analysis. The amorphous chalcogenide materials tend to be flexible and can move (constrict volume, expand volume, pull away from interfaces) which could have a ripple effect around any ions within the material or provide alternative electron conduction pathways as a function of temperature. In this work, the switching properties at a given temperature were studied, not how a pre-programmed property changed as a function of temperature.

Despite the stated concerns, several observations can be made from the DC switching data as a function of temperature. The lower initial resistance of the Cu devices at 300 K in Figure 5a (especially compared to the R_E_ values, Figure 5c) indicate that the Cu devices have experienced Cu diffusion into the active layer over time while stored at ambient room temperature. This limits the Cu device data retention. This is not the case for the Ag devices. This conclusion can be reached for three reasons: (1) the lower initial resistance of the Cu device; (2) since both the Ag and Cu devices are pristine in the initial resistance measurement, the active layers should be the same and give the same Ri throughout the temperature measurement range; and (3) the erased resistance of the Cu device is the same as the Ag device when they are at higher temperatures (Figure 5c), as expected if excess Ag and Cu have been removed from the channel during the reverse potential sweep. 

The Cu migration may be responsible for the well-behaved switching of the Cu devices at low temperatures during the Write 1 sweep (Figure 2c). Since this diffused Cu may be removed during an Erase sweep, it could account for the worse switching observed in the I–V curves for the Write 2 sweeps (Figure 2d) compared to the Write 1 I–V curves. 

The Cu devices survive switching at temperatures down to 6 K. Figure 4 shows the Cu device Erase I-V sweeps and it is clear that devices erase at all temperatures. This is supported by the R_E_ data shown in Figure 5c. In addition to the robustness of the Cu-based SDC device, a Cu-silica memristor was also shown to survive operation at 4 K [11].

The Ag device I-V curves have three distinct temperature transitions at 230 K, 210 K, and 140 K in which the ability of the device to write varies. The R_W2_ and R_E_ data for the Ag devices indicate that below 150 K these devices are damaged if they are operated. This is not the case for the Cu devices. However, it is notable that the Cu devices have higher Write resistances when operated at T > 200 K (Figure 5b,d). It is interesting to note that the effect of temperature on R_W1_ and R_W2_ for Ag and Cu devices is opposite. Taking R_W1_ as an example, above 200 K, the slope of the ln(1/R_W1_) vs 1000/T plot (Figure 5b) is negative for the Ag devices, but positive for the Cu devices. Between temperatures of 200 K and 150 K, the Ag devices exhibit high resistance trough with little resistance change; the Cu devices exhibit a hill-like peak of decreasing resistance. A similar, but less pronounced, effect is seen for R_W2_ in Figure 5d.

The Ag device R_W1_ resistances are higher than those for the Cu devices at temperatures between 200K and 150 K (Figure 5b) since within this range, the Ag devices do not switch out of the high resistance state. When the temperature is further reduced to a range where T < 150 K, the devices ‘break’ (the exact ‘breaking’ temperature is unknown). As previously mentioned, Ag devices switched at these temperatures are damaged. The formation of a Sn-Ag alloy at the lower temperatures might be the cause of the inability to erase devices that have been written at those temperatures.

The write threshold voltages for each device type as a function of temperature are in Figure 6. The Write 2 threshold voltage is the same between both device types (Figure 6b). This seems logical if a channel is formed on the first write and used for small movement of mobile ions within the channel during subsequent programming events. Note again that there is a divergence in device response between Ag and Cu near 150 K. This is likely due to differences in Ag or Cu participation in the chemical reaction of channel formation and Ag or Cu storage in the SnSe layer that become relevant when higher voltages are applied. In addition, the first and second write thresholds exhibit an exponential dependence on temperature between 300 K and 150 K. This exponential behavior has been attributed to the collective motion of carriers in metal-insulator transition studies, for example two-terminal VO_2_ devices [45]. In the SDC device case, this could correspond to the collective motion of mobile ions, or to the formation of a Sn-Ag alloy (or Sn-Cu alloy) [39,40].

Sinusoidal excitation (Figure 7) and pulse studies (Figure 8) can offer insights into ion movement and channel formation for each device type. During sinusoidal excitation, the Cu devices show flattened hysteresis lobes at an input signal frequency of 100 Hz, whereas Ag devices achieve flattened lobes at approximately 10 kHz. The significance of this is still not understood, however, the two device types demonstrate differences in switching speed. The possibility of using the frequency at which lobes flatten as a predictor of device switching speed would offer a simple way to predict device speed prior to more complicated pulsed measurements. 

## 5. Conclusions

The electrical behavior of the Ag and Cu-based memristors over a large temperature range is complex. The factors that contribute to device operation are varied and include the effects of temperature on the active Ge_2_Se_3_ material layer’s flexibility, the chemical reaction involved in formation of the self-directed channels, and the redox reactions of Ag, Cu, and Sn from the SnSe layer. The Ag-based devices appear to be damaged when operated at low temperatures. However, it is possible this is due only to the increased potential applied during the Write sweeps at lower temperatures and a resultant alloy formation with Sn. Further work is underway to quantify the effects of the interaction between the SnSe layer and Ag and Cu during device operation and to understand any potential alloy formation between Sn and Ag or Cu. 

Interestingly, the Cu-based devices showed a migration of Cu through the active layer over time. This migration is detrimental for long term data storage since the device will lose any programmed data state. The Ag-based devices did not exhibit this response.

The Ag-based devices appear to exhibit faster pulsed programming switching during the first programming Write cycle. Faster response time of the Ag devices was also observed in the sinusoidal excitation measurements where the Ag-devices exhibited a flattening of the characteristic memristor hysteresis loop at 10 kHz, whereas the Cu-based devices exhibited flattening at 100 Hz. 

The formation of the self-directed channels as a function of the SnSe layer should be studied through the replacement of Sn within that layer, with different metals. It is possible that any alloy formation between the mobile ion and the metal from the metal chalcogenide layer could have a significant impact on device performance, and be a method of selected device performance tuning. If Sn-Ag alloy is responsible for the device damage when higher voltages at low temperatures, it may be possible to change the metal in the metal-chalcogenide layer to one less likely to alloy with Ag.

## Figures and Tables

**Table 1:** I–V sweep resistance measurement descriptions.

Resistance	Resistance Measurement Sweep
Initial, R_i_	+20 mV on Write 1
First Write, R_W1_	−20 mV on Erase
Erased, R_E_	+20 mV on Write 2
Second Write, R_W2_	+20 mV on Read

## Supplementary Materials

The following are available online at https://www.mdpi.com/2072-666X/10/10/663/s1, Figure S1, a data retention plot for Ag-based SDC devices. Figure S2, Ag-based device switching voltage vs sweep rate.
