# Supplementary material for: Comparison of the Electrical Response of Cu and Ag Ion-Conducting SDC Memristors Over the Temperature Range 6 K to 300 K
Source: Micromachines (Basel). 2019 Sep 30;10(10):663. doi: 10.3390/mi10100663 (PMC6843820; doi:10.3390/mi10100663)
Supplement: Supplementary file 1 [file micromachines-10-00663-s001.pdf]

micromachines

Supplemental Material

Comparison of the Electrical Response of Cu and Ag Ion-Conducting SDC Memristors Over the Temperature Range 6 K to 300 K

Kolton Drake, Tonglin Lu, Md. Kamrul H. Majumdar and Kristy A. Campbell *

Department of Electrical and Computer Engineering, Boise State University, Boise, ID 83725, USA
* Correspondence: kriscampbell@boisestate.edu; Tel.: +1-208-426-5968

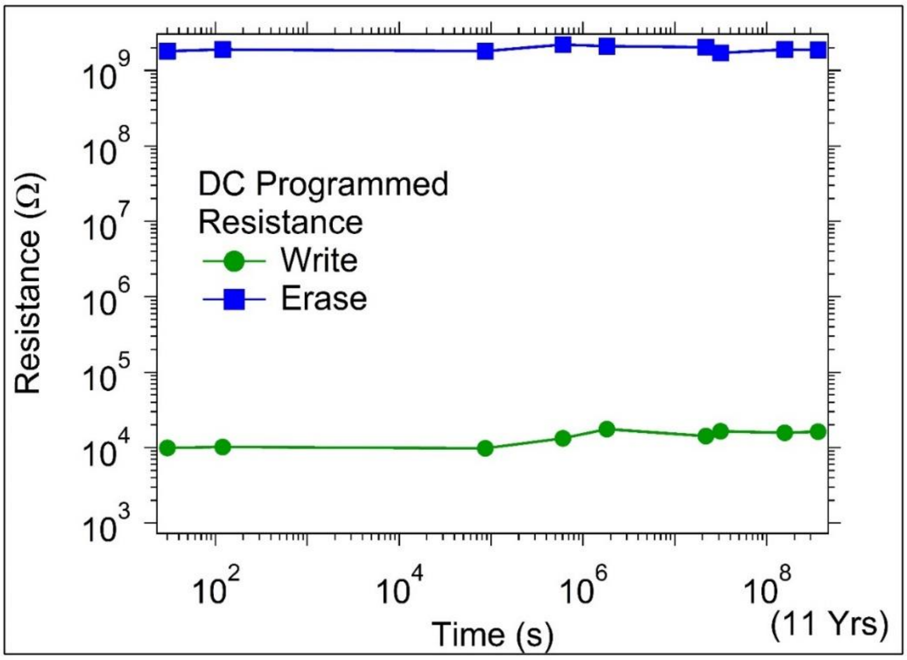

Figure 1. Data retention of Ag-based SDC memristors over 11 years. 20 devices are in a written state and 20 devices in an erased state. Measurements are made periodically to investigate state drift.

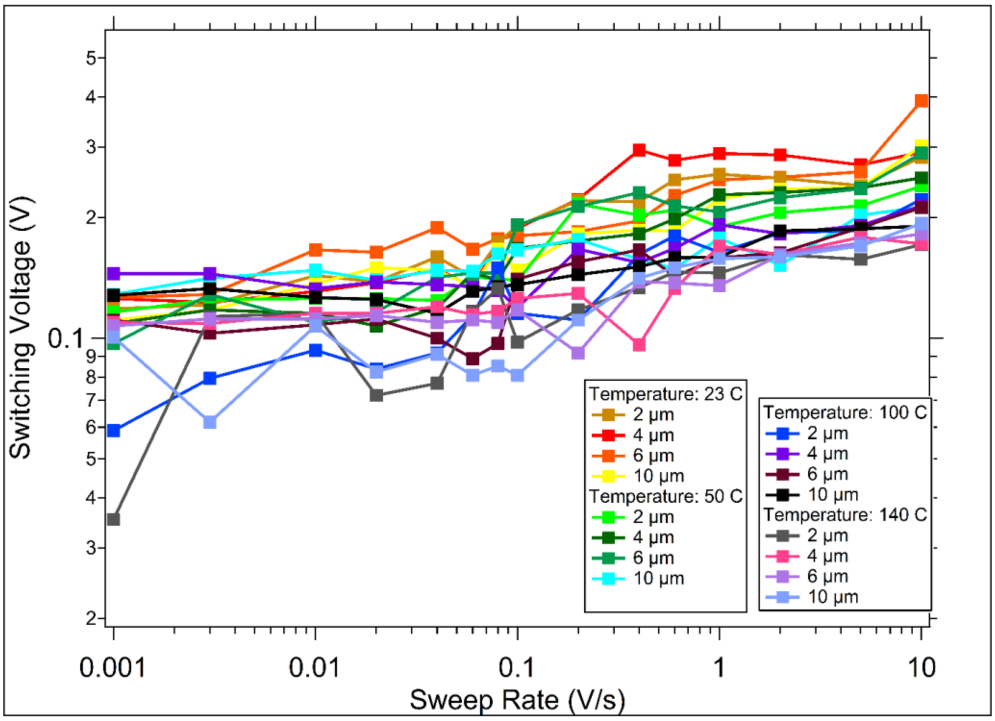

Figure 2. Ag-based device switching voltage as a function of DC sweep rate over the temperature range of ~300 K to 413 K.
